# Clinical and immunological characteristics for BK polyomavirus‐associated nephropathy after kidney transplantation

**DOI:** 10.1002/iid3.956

**Published:** 2023-08-28

**Authors:** Tanaya Siripoon, Nopporn Apiwattanakul, Pannawat Mongkolrattanakul, Chutatip Tongsook, Nattawut Unwanatham, Suradej Hongeng, Surasak Kantachuvesiri, Jackrapong Bruminhent

**Affiliations:** ^1^ Division of Infectious Diseases, Department of Medicine, Faculty of Medicine Ramathibodi Hospital Mahidol University Bangkok Thailand; ^2^ Department of Clinical Tropical Medicine, Faculty of Tropical Medicine Mahidol University Bangkok Thailand; ^3^ Division of Infectious Diseases, Department of Pediatrics, Faculty of Medicine Ramathibodi Hospital Mahidol University Bangkok Thailand; ^4^ Division of Nephrology, Department of Medicine, Faculty of Medicine Ramathibodi Hospital Mahidol University Bangkok Thailand; ^5^ Division of Virology, Department of Pathology, Faculty of Medicine Ramathibodi Hospital Mahidol University Bangkok Thailand; ^6^ Department of Clinical Epidemiology and Biostatistics, Faculty of Medicine Ramathibodi Hospital Mahidol University Bangkok Thailand; ^7^ Division of Hematology and Oncology, Department of Pediatrics, Faculty of Medicine Ramathibodi Hospital Mahidol University Bangkok Thailand; ^8^ Ramathibodi Excellence Center for Organ Transplantation, Faculty of Medicine Ramathibodi Hospital Mahidol University Bangkok Thailand

**Keywords:** BK polyomavirus, innate immunity, natural killer cell, natural killer T cell, renal transplantation

## Abstract

**Introduction:**

BK polyomavirus (BKPyV)‐associated nephropathy (BKPyVAN) can cause a significant risk of allograft impairment after kidney transplantation (KT). Intact BKPyV‐specific immunity is associated with viral containment. This study investigated BKPyV‐specific immunological factors among KT recipients.

**Methods:**

This prospective study in a single transplant center from January 2019 to August 2019 assessed associations between clinical and immunological characteristics, with a focus on BKPyV‐cell‐specific immunity and BKPyVAN, among KT recipients aged ≥15 years. The numbers of interferon‐gamma (IFN‐γ)‐producing CD4^+^ T, CD8^+^ T, natural killer (NK), and natural killer T (NKT) cells were measured after stimulation with large T antigen and viral capsid protein 1 (VP1).

**Results:**

In total, 100 KT recipients were included (mean age ± SD, 42 ± 11 years); 35% of the recipients were female patients, and 70% had received induction immunosuppressive therapy. The 1‐year cumulative incidence of high‐level BKPyV DNAuria (possible BKPyVAN) and (presumptive BKPyVAN) was 18%. Among 40 patients with immunological factor data, pre‐KT %NK cells (hazard ratio [HR], 1.258; 95% confidence interval [CI], 1.077–1.469; *p* = .004) and %VP1‐specific NK cells (HR, 1.209; 95% CI, 1.055–1.386; *p* = .006) were factors independently associated with possible and presumptive BKPyVAN. KT recipients with possible and presumptive BKPyVAN were more likely to exhibit significant mean coefficients of %NK, %VP1‐specific NK, and %NKT cells at 1 month after KT than before KT (all *p* < .05).

**Conclusion:**

Individuals with nonspecific and VP1‐specific NK cells before KT and increasing numbers of these cells after KT may be at risk for high‐level BKPyV DNAuria and presumptive BKPyVAN. Further studies are needed to determine the utility of BKPyV‐specific innate immune surveillance in predicting the occurrence of BKPyVAN.

## Graphical abstract

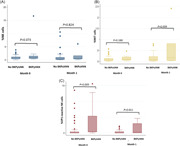

## 1 INTRODUCTION

BK polyomavirus (BKPyV) infection can cause BKPyV‐associated nephropathy (BKPyVAN) in kidney transplantation (KT) recipients, resulting in allograft dysfunction. Immunosuppressive drugs are important for efforts to prevent allograft rejection. However, immunosuppression can lead to BKPyV reactivation, followed by BKPyV DNAuria with potential progression to DNAemia and BKPyVAN.^1^ In addition to direct virus‐induced allograft impairment, the reduction of immunosuppressive drugs to manage BKPyVAN can result in allograft rejection.^2^, ^3^, ^4^ Previous studies have focused on the restoration of BKPyV‐specific immune responses in KT recipients.^3^, ^5^, ^6^ A lack of BKPyV‐specific humoral immunity (e.g., antibodies) or BKPyV‐specific cell‐mediated immunity (e.g., T‐cell responses before transplantation) is associated with BKPyV reactivation after KT.^7^ Adequate BKPyV‐specific T‐cell immunity, both before KT and during the early post‐KT period, is reportedly associated with clearance of BKPyV.^7^ Furthermore, aspects of innate immunity, such as the roles of natural killer (NK) and dendritic cells in controlling BKPyV reactivation, have not been explored; the underlying mechanism remains unclear.^8^, ^9^, ^10^, ^11^ Here, we assessed the incidences of BKPyV DNAuria, DNAemia, and BKPyVAN, as well as the associations of NK and NKT cell‐specific immune responses with BKPyVAN, within 1 year after KT. We hypothesized that the incidences of BKPyV DNAuria (possible BKPyVAN) and proven BKPyVAN would be comparable to the incidences in previous studies.^12^ We also hypothesized that NK and natural killer T (NKT) cell‐specific immune responses would be associated with BKPyV DNAuria, DNAemia, and BKPyVAN in our cohort.

## 2 METHODS

### 2.1 Study design

This prospective cohort study was conducted at a single transplant center. All patients aged ≥15 years who underwent KT from January 2019 to August 2019 were enrolled. The primary endpoint was the 1‐year cumulative incidence of BKPyV DNAuria (possible BKPyVAN), BKPyV DNAemia (presumptive BKPyVAN) and proven BKPyVAN. The secondary endpoints were DNAuria and BKPyVAN risk factors and outcomes. Finally, the exploratory outcomes were the associations of BKPyV‐specific immunity with BKPyV DNAuria and presumptive BKPyVAN. Sample size analysis using a one‐sample proportion test showed that, for 80% power in assessing the incidences of BKPyV DNAuria and presumptive BKPyVAN, 80 patients were required. Possible and proven BKPyVAN incidences of 20% (reported in previous studies^2^, ^3^, ^4^, ^13^) were used in this calculation. Considering an expected dropout rate of 10%, we enrolled 100 patients. Allograft type, human leukocyte antigen match, panel reactive antibodies, and immunosuppressant type were recorded, along with clinical risk factors, immunological risk factors, and the outcomes of BKPyV DNAuria, BKPyV DNAemia, and proven BKPyVAN. Preemptive measurements of urine BKPyV DNA loads were conducted before KT and at 1, 2, 3, 6, 9, and 12 months (±1 month) after KT (Supporting Information: Figure S1). Both blood and urine specimens were collected; for KT recipients with BKPyV DNAuria, assessments of archived plasma were conducted. Kidney biopsies were performed in accordance with nephrologist preferences. Because of laboratory constraints, we included all participants with evaluable immunological profiles in the initial analysis of BKPyV‐specific immune cells. We investigated cluster of differentiation (CD)4^+^ T, CD8^+^ T, NK, and NKT cells that produced interferon‐gamma (IFN‐γ) after stimulation with BKPyV‐specific antigens before KT and at 1‐month post‐KT, among subsets of cells from KT recipients; the results are reported as percentages. The study protocol was approved by the Institutional Review Board of the Faculty of Medicine at Ramathibodi Hospital, Mahidol University, Bangkok, Thailand (approval no. ID 10‐61‐11).

### 2.2 Definition

BKPyVAN was defined in accordance with the American Society of Transplantation Infectious Diseases Community of Practice guidelines (Supporting Information: Table S1).^3^, ^5^ Possible BKPyVAN was defined as BKPyV DNAuria >7 log10 copies/mL, presumptive BKPyVAN was defined as BKPyV DNAemia >4 log10 copies/mL, and proven BKPyVAN was defined via biopsy showing viral cytopathic changes, inflammatory infiltrates, tubulitis, or more than mild interstitial fibrosis/tubular atrophy. Immunohistochemistry was conducted to assess monoclonal antibody staining of polyomavirus simian virus 40 large T antigen (LT).^3^, ^14^ Intracellular cytokine assays using VP1 and LT were performed to evaluate IFN‐γ‐producing CD4^+^ T cells, CD8^+^ T cells, NK cells, and NKT cells before and at 1 month ± 7 days after transplantation.

### 2.3 Analysis of BK‐specific T and NK cells

Peripheral blood mononuclear cell preparation for the quantification of virus‐specific T and NK cells was performed as previously described.^15^ Briefly, heparinized blood was subjected to density gradient centrifugation using polysucrose‐sodium diatrizoate (Lymphoprep™) purchased from Axis‐Shield PoC AS. After separation, the cells were thoroughly washed with phosphate‐buffered saline and resuspended in RPMI medium (Gibco) supplemented with 10% fetal calf serum (Gibco), then seeded in 96‐well tissue culture plates (Corning Costar) at 1 × 10^5^ cells/200 µL in each well. Subsequently, individual virus‐specific antigens were added to each well. The BK antigens used were overlapping peptide pools (PepMix™ peptide pools) containing the LT and VP1 antigens, all purchased from JPT Peptide Technologies. In each overlapping peptide pool, the final concentration was 1 mg/mL. Wells containing only cells (no added antigen) served as negative controls. The plates were incubated at 37°C with 5% CO_2_ in humidified air for 12 h before the addition of brefeldin A (eBioscience) to each well. The plates were incubated for an additional 6 h before cell fixation and staining, as described below.

Next, the cells were fixed with 1% formaldehyde (Sigma‐Aldrich) for 15 min at room temperature. After fixation, the cells were washed, resuspended in 0.5% saponin (Sigma‐Aldrich), and incubated at room temperature for 15 min; they were stained with fluorescence‐tagged antibodies suspended in 0.5% saponin. The antibody cocktail consisted of a 1:100 dilution of FITC‐CD3, PE‐CD56, APC‐CD4, eFluor780‐CD8, and PE‐Cy7 IFN‐γ. All antibodies were procured from eBioscience. Cells were incubated in the antibody cocktail for 30 min at 4°C, then subjected to flow cytometry analysis.

The percentages of virus‐specific CD4^+^ T cells (CD3^+^CD4^+^), CD8^+^ T cells (CD3^+^CD8^+^), NK cells (CD56^+^), and NKT cells (CD3^+^CD56^+^) reacting with each antigen were calculated as the proportion of IFN‐γ‐producing cells among all CD4^+^ T cells, CD8^+^ T cells, NK cells, or NKT cells in the presence of antigen after subtracting the proportion of IFN‐γ‐producing cells in negative control samples. Data were analyzed by using FlowJo v.10 software (FlowJo).

### 2.4 Statistical analyses

The cumulative incidence of BKPyVAN was estimated using Kaplan–Meier analysis. A Cox proportional hazards model was used to analyze clinical and immunological risk factors for BKPyV DNAuria. Categorical variables are presented as absolute numbers and relative frequencies; continuous variables are presented as means with standard deviations. The percentages of IFN‐γ‐producing CD4^+^ T, CD8^+^ T, NK, and NKT cells before and 1 month after KT were compared by mixed linear regression analysis. *p* Values < 0.05 were considered statistically significant. Statistical analyses were conducted, and linear plots of BKPyV‐specific immune cells were constructed, using Stata v.16 statistical software (StataCorp).

## 3 RESULTS

### 3.1 Baseline characteristics

**Figure 1:**
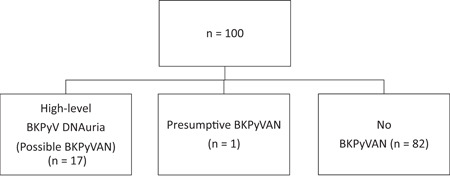
Flowchart of patient selection for this study. BKPyV, BK polyomavirus; BKPyVAN, BKPyV‐associated nephropathy.

We enrolled 100 KT recipients (Figure 1). Some patients were analyzed until censoring because they died of causes other than BKPyVAN (*n* = 2), had allograft loss unrelated to BKPyVAN (*n* = 5), or were lost to follow‐up (*n* = 3). Clinical characteristics are shown in Table 1. The mean age ± standard deviation was 42 ± 11 years. Thirty‐five patients (35%) were female. The most common etiologies of end‐stage kidney disease were unknown (69%), glomerulonephritis (15%), and diabetic nephropathy (7%). Sixty‐three patients (63%) received a deceased donor allograft, and 70 patients (70%) received induction immunosuppressive therapy (3% anti‐thymocyte globulin [ATG] and 67% basiliximab). Medications for maintenance immunosuppression included tacrolimus (81%), cyclosporin (19%), mycophenolate mofetil (56%), and prednisolone (99%).

**Table 1:** Clinical characteristics of 100 kidney transplant recipients.

Characteristics	*N* (%)^a^
Female sex	35 (35)
Age (years), mean ± SD	42 ± 11
ESKD etiology	
Diabetic nephropathy	7 (7)
Hypertension	5 (5)
Glomerulonephritis	15 (15)
Unknown	69 (69)
Others	4 (4)
Type of transplant	
DDKT	63 (63)
LRKT	37 (37)
Cytomegalovirus serostatus	
D+/R+	96 (96)
D−/R+	1 (1)
D+/R−	2 (2)
D−/R−	1 (2)
Terminal creatinine (median [IQR])	0.85 (0.52–2.51)
Human leukocyte antigen mismatch	
0	11 (11)
1–3	74 (74)
4–6	15 (15)
Panel reactive antibody	
1–10	91 (91)
11–50	4 (4)
>50	5 (5)
Induction therapy^b^	
Basiliximab	67 (67)
Anti‐thymocyte globulin	3 (3)
None	30 (30)
Maintenance therapy	
Tacrolimus	81 (81)
Cyclosporin	19 (19)
Mycophenolate sodium	43 (43)
Mycophenolate mofetil	56 (56)
Prednisolone	99 (99)

Abbreviations: DDKT, deceased donor kidney transplantation; ESKD, end‐staged kidney disease; IQR, interquartile range; PRA, panel‐reactive antibody.

^a^ Data are shown as number (%) unless otherwise indicated.

^b^ Participants receiving the indicated immunosuppressive agents were counted.

### 3.2 Incidences of possible and presumptive BKPyVAN

**Figure 2:**
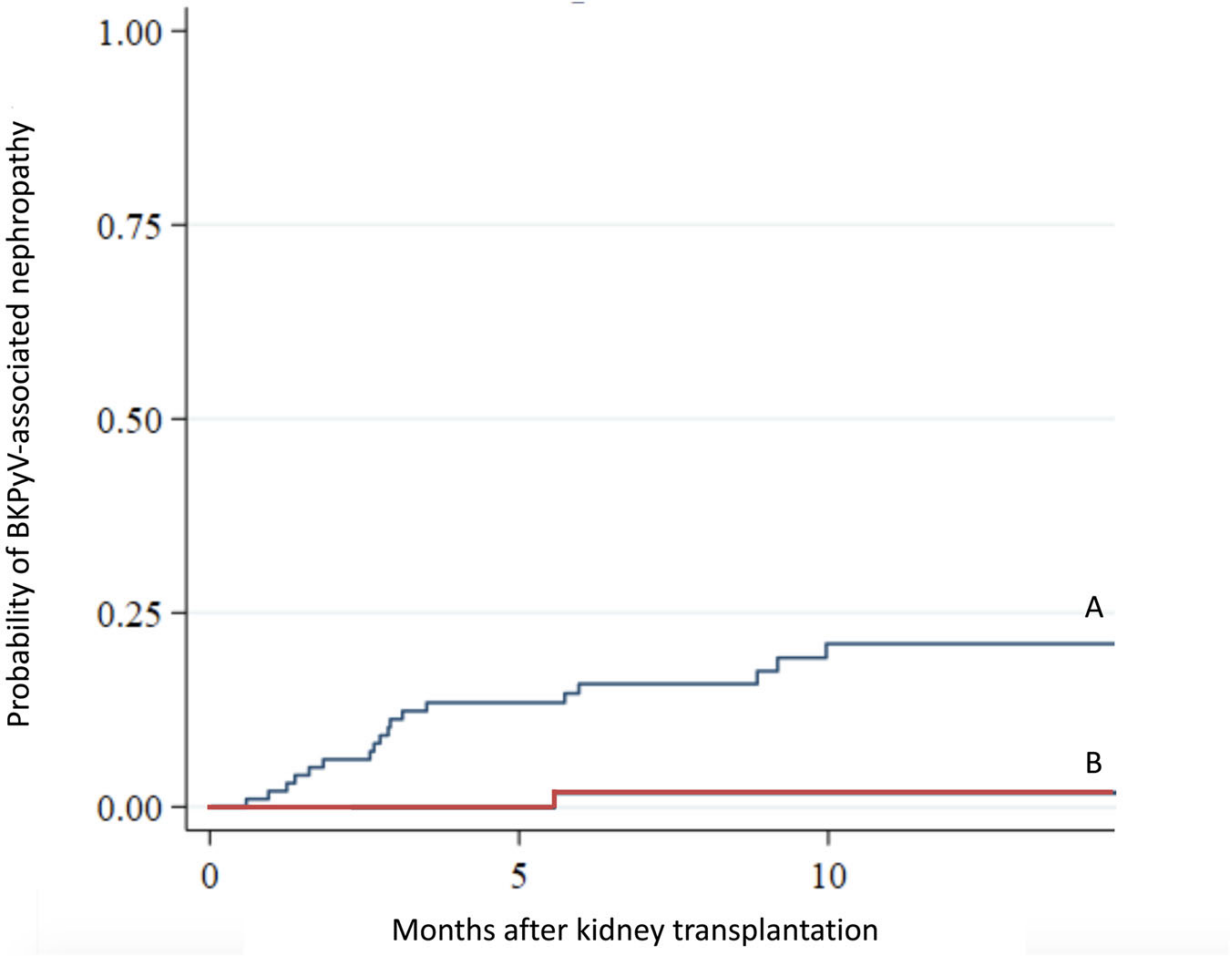
Cumulative incidences of possible and presumptive BKPyV‐associated nephropathy. (A, B) Kaplan–Meier plots of possible (A) and presumptive (B) BKPyV‐associated nephropathy within 1 year after KT. BKPyV, BK polyomavirus; KT, kidney transplantation.

The cumulative incidences of BKPyV DNAuria (possible BKPyVAN) and BKPyV DNAemia (presumptive BKPyVAN) were 17/100 (17%) and 1/100 (1%), respectively (Figure 2A,B). The incidence rates of possible and presumptive BKPyVAN were 20.999/100 and 1.166/100 person‐months, respectively. No patients had proven BKPyVAN.

### 3.3 Risk factors for possible and presumptive BKPyVAN

Univariate analysis revealed that only one factor was significantly associated with possible and presumptive BKPyVAN: underlying diabetic nephropathy (hazard ratio [HR] 7.548; 95% confidence interval [CI], 2.257–25.246; *p* = .001; Table 2).

**Table 2:** Factors associated with possible and presumptive BKPyVAN among kidney transplant recipients.

Factors	Univariate analysis			Multivariate analysis		
	HR	95% CI	*p*	HR	95% CI	*p*
Female sex	0.958	0.354–2.594	.933			
Age (per 1 year)	1.002	0.961–1.044	.924			
Body mass index (per 1 kg/m^2^)	0.998	0.896–1.111	.967			
ESKD etiology						
Diabetic nephropathy	7.548	2.257–25.246	.001			
Glomerulonephritis	3.355	1.094–10.290	.034			
Others	2.868	0.356–23.098	.322			
Unknown	Ref.					
DDKT	1.195	0.448–3.186	.722			
Creatinine on day of discharge (per 1 mg/dL)	0.588	0.197–1.756	.342			
Human leukocyte antigen mismatch (per 1 mismatch)	1.094	0.424–2.823	.852			
PRA (%)						
11–50	3.647	0.829–16.070	.087			
>51	3.413	0.772–15.101	.106			
1–10	Ref.					
Induction therapy						
Basiliximab	2.308	0.663–8.038	.189			
Anti‐thymocyte globulin	4.885	0.507–47.038	.170			
None	Ref.					
Pre‐KT BKPyV‐specific immunity^a^						
%NK cells	1.127	0.989–1.284	.073	1.26	1.077–1.469	.004
%LT‐specific NK cells	0.860	0.527–1.404	.546			
%VP1‐specific NK cells	1.201	1.058–1.363	.005	1.209	1.055–1.386	.006
%NKT cells	17.351	0.248–1215.659	.188			
%LT‐specific NKT cells	0.860	0.525–1.407	.547			
%VP1‐specific NKT cells	1.001	0.989–1.126	.993			
1‐month BKPyV‐specific immunity^b^						
%NK cells	1.033	0.775–1.378	.824			
%LT‐specific NK cells	1.097	0.562–2.145	.785			
%VP1‐specific NK cells	2.009	1.171–3.446	.011			
%NKT cells	4.876	1.482–16.050	.009			
%LT‐specific NKT cells	0.700	0.275–1.781	.454			
%VP1‐specific NKT cells	0.898	0.603–1.338	.598			

Abbreviations: BKPyV, BK polyomavirus; CI, confidence interval; HR, hazard ratio; LT, large T antigen; NKT, natural killer T; VP1, viral capsid protein 1.

^a^ Sixty‐five evaluable patients.

^b^ Forty evaluable patients.

**Figure 3:**
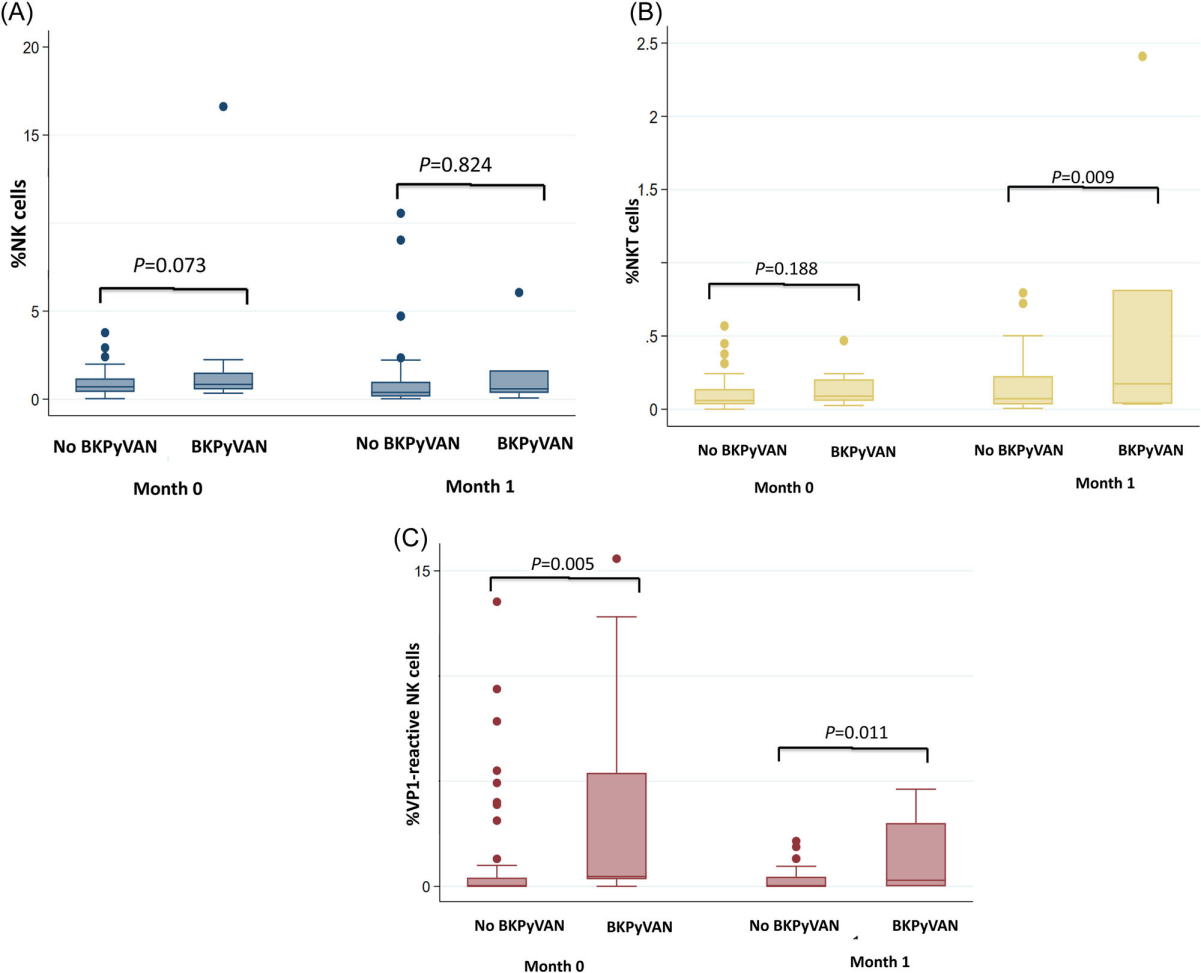
Pre‐KT and 1‐month post‐KT percentages of NK (A), NKT (B), and VP1‐reactive NK cells among patients with and without possible and presumptive BKPyVAN. BKPyVAN, BKPyV‐associated nephropathy; KT, kidney transplantation; NK, natural killer; NKT, natural killer T; VP1, viral capsid protein 1.

There were 40 KT recipients with available BKPyV‐specific immune cell measurements before and 1 month after KT (Figure 3A–C). Univariate analysis revealed that three factors were significantly associated with possible and presumptive BKPyVAN: pre‐KT %VP1‐specific NK cells (hazard ratio [HR], 1.201; 95% confidence interval [CI], 1.058–1.363; *p* = .005), 1‐month post‐KT %VP1‐specific NK cells (HR, 2.009; 95% CI, 1.171–3.446; *p* = .011), and 1‐month post‐KT %NKT cells (HR, 4.876; 95% CI, 1.482–16.050; *p* = .009). Pre‐KT %NK cells showed a tendency toward association with BKPyVAN (HR, 1.127; 95% CI, 0.989–1.284; *p* = .073). In multivariate analysis, pre‐KT factors independently associated with possible and presumptive BKPyVAN were pre‐KT %VP1‐specific NK cells (HR, 1.201; 95% CI, 1.058–1.363; *p* = .005), and pre‐KT %NK cells (HR, 1.258; 95% CI, 1.077–1.469; *p* = .004), as shown in Table 2.

### 3.4 BKPyV‐specific immune responses over time

**Figure 4:**
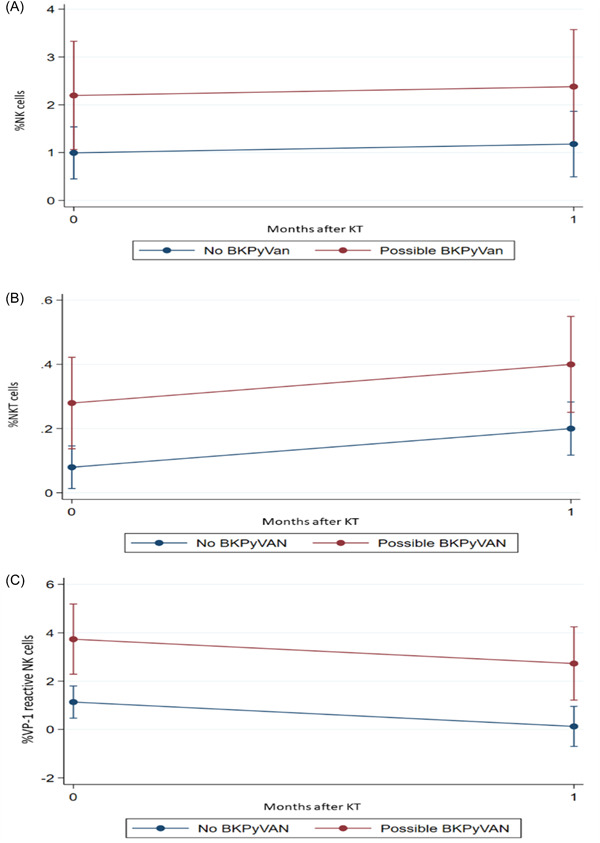
Mean percentages of NK (A), NK (B), and VP1‐specific NK cells (C) before and 1 month after KT among patients with possible BKPyVAN (red) and without possible BKPyVAN (blue). BKPyVAN, BKPyV‐associated nephropathy; KT, kidney transplantation; NK, natural killer; NKT, natural killer T; VP1, viral capsid protein 1.

Mixed linear regression analysis of BKPyV‐specific immunity in 40 patients was assessed in relation to time after transplantation and possible BKPyVAN (Table S2). Five of the 40 patients (12.5%) developed BKPyV DNAuria (data not shown). On average, the coefficients of %LT‐specific CD4^+^ T cells (0.007; 95% CI, 0.001–0.013; *p* = .018) and %NKT cells (0.120; 95% CI, 0.025–0.215; *p* = .013) significantly increased over time from pre‐KT to 1‐month post‐KT, whereas the coefficient of %VP1‐specific NK cells (−1.007; 95% CI, −1.934 to −0.080; *p* = .033) significantly decreased over time. Among patients with BKPyVAN, the mean coefficients of %VP1‐specific NK cells (2.602; 95% CI, 1.083–4.121; *p* = .001), %NKT cells (0.199; 95% CI, 0.051–0.348; *p* = .008), and %NK cells (1.202; 95% CI, 0.033–2.371; *p* = .044) cells significantly increased over time from pre‐KT to 1‐month post‐KT. Linear plots of those BKPyV‐specific immune cells in BKPyVAN‐free KT recipients, before and 1 month after transplantation, are presented in Figure 4. Analyses of laboratory‐developed intracellular cytokine assays measuring the percentages of IFN‐γ‐producing CD4^+^ T cells, CD8^+^ T cells, NK cells, and NKT cells after incubation with LT and VP1 are shown in Supporting Information: Figure S2; PE‐CD56 staining results and findings in control cells are shown in Supporting Information: Figure S3.

### 3.5 Outcomes

At 1 year, 27 (27%) patients had developed allograft dysfunction, and 2 (2%) had died of an undetermined etiology that was considered unrelated to BKPyVAN. Additionally, five patients had experienced allograft loss; four of these losses were considered unrelated to BKPyVAN. Seventeen patients with BKPyV DNAuria were subjected to analysis of plasma BKPyV using a sample collected on the same date; they received treatment for possible BKPyVAN based on the clinician's assessment of the findings. Furthermore, one patient developed presumptive BKPyVAN and received treatment for BKPyVAN. The patient later developed allograft rejection and proven BKPyVAN, which led to allograft loss at 12‐month post‐KT. Two patients received treatment for possible BKPyVAN and subsequentially developed proven BKPyVAN without allograft loss. One patient with possible BKPyVAN was diagnosed with respiratory syncytial virus bronchitis and *Listeria monocytogenes* bacteremia during the diagnosis of possible BKPyVAN.

## 4 DISCUSSION

This study investigated immunological factors, including nonspecific and BKPyV‐specific T‐cell, NK‐cell, and NKT‐cell immunity, in KT recipients who experienced BKPyVAN within 1 year after KT. The results showed that individuals who had underlying diabetic nephropathy and BKPyV‐specific innate immunity before transplantation, indicated by the presence of BKPyV‐specific NK cells that secreted IFN‐γ after stimulation with VP1 antigen, were more likely to develop BKPyV DNAuria within 1 year after KT. Additionally, the proportion of VP1‐specific NK cells in KT recipients showed a tendency to increase after transplantation, as indicated by BKPyV DNAuria, despite post‐transplant immunosuppression.

The incidences of BKPyV DNAuria and BKPyV DNAemia in our KT recipients were comparable to the findings in previous studies.^12^, ^16^ Two retrospective studies reported that the prevalence of BKPyVAN was 10%–12% among KT recipients with similar demographic characteristics. However, the rate of possible BKPyVAN in our study was relatively high (20%), which could be explained by proactive monitoring to detect early BKPyV DNAuria. Furthermore, none of our patients had proven BKPyVAN at the time of initial diagnosis, presumably because we conducted early optimization of immunosuppression; this finding considerably differs from the results of studies, in which 6.4%–8.6% of patients had proven BKPyVAN.^12^, ^16^ Furthermore, we reaffirmed the importance of underlying diabetes as a predictive factor for BKPyV DNAuria.^3^

Because no effective anti‐BKPyV agent is available, immunosuppressive therapy modification is a key management approach for the restoration of immune function. Therefore, BKPyV‐specific immune surveillance may be a useful tool for posttreatment prevention and disease monitoring.^1^, ^7^, ^17^ Thus far, virus‐specific T‐cell immunity has played an important role in controlling viral replication in both solid organ and hematopoietic stem cell transplant recipients.^18^ There is evidence that the absence of virus‐specific T‐cell immune responses increases the risk of adenovirus, BKPyV, and cytomegalovirus infections.^18^, ^19^, ^20^ Although most studies have investigated the role of cytomegalovirus‐specific T‐cell immunity and their findings have been implemented in clinical practice, there is limited data regarding BKPyV‐specific immunity.^21^, ^22^

Adaptive immune responses, particularly T‐cell responses, constitute the main mechanism for controlling BKPyV infection. Increases in CD4+ and CD8^+^ T cells were observed in patients with DNAemia who achieved clearance of BKPyV.^7^ LT‐specific CD8^+^ T cells and VP1‐specific CD4^+^ BKPyV‐specific T cells may be particularly important.^8^ Furthermore, a recent study showed that KT recipients with BKPyVAN tended to exhibit recovery of LT‐specific CD4^+^ T‐cell responses after their immunosuppression treatment had been optimized.^19^

We speculate that increased pre‐transplant BKPyV‐specific innate immunity leads to greater percentages of NK and VP1‐specific NK cells after KT, which may be associated with BKPyVAN. The innate immune system has key roles in suppressing viral replication and activating adaptive immunity to eradicate viruses.^23^ Inflammatory NK cell antiviral responses consist of interactions between immunoglobulin‐like receptors on NK cells and major histocompatibility complex class I molecules on virus‐infected cells, which influence their sensitivity to lysis by NK cells.^24^ NK cells eradicate BKPyV by inducing apoptosis in infected cells through antibody‐dependent cellular cytotoxicity.^25^ Ischemic injury to an allograft may reactivate BKPyV in that allograft, thereby triggering the innate immune system. NK cells recognize virus‐infected cells through the downregulation of major histocompatibility complex class I receptors and inhibitory receptors, as well as the upregulation of activator molecules.^23^ There is evidence that the proportion of activating NK‐cell immunoglobulin‐like receptors is significantly smaller in patients with BKPyVAN than in healthy controls.^8^, ^23^, ^26^ NKT cells have recently gained attention as a bridge between the innate and adaptive immune systems. In a herpes virus infection model, NKT cells functioned as T‐helper cells and as cytotoxic T cells.^27^ NKT cells can promote early antibody‐based immunity after viral infection by enhancing B‐cell antibody responses.^28^ Previous reviews have suggested that innate immunity plays a crucial role in BKPyVAN; however, our results contradict this notion. Thus, there is a need to explore the role and mechanism of innate immunity in BKPyVAN, particularly concerning NK cells, dendritic cells, neutrophils, eosinophils, Toll‐like receptors, and chemokines.

To our knowledge, only a few studies have identified immunological factors associated with BKPyV infection. A notable strength of the present study is that it explored an early stage of BKPyV DNAuria (possible BKPyVAN) and early immune responses, such as the innate immunity represented by BKPyV‐specific NK cell responses. We also emphasized the importance of both innate and adaptive BKPyV‐specific immunity in the development of BKPyV DNAuria. We believe that our findings will address knowledge gaps in this field.^3^ Our findings highlight the need for a post‐KT BKPyV screening surveillance system for KT recipients, considering the limited conventional treatment options. Additionally, BKPyV‐specific T‐cell and antibody responses may serve as adjunct markers of viral clearance, offering guidance for clinicians who encounter this infection. However, this study also had some limitations. First, although urine BKPyV DNA load can be used to screen for BKPyVAN,^3^, ^29^ it has a less obvious relationship with proven BKPyVAN and possibly lower cost‐effectiveness, compared with plasma BKPyV DNA load.^30^ Second, we did not adjust for BKPyV‐specific humoral immunity, which is reportedly associated with BKPyVAN. Third, our study was relatively small, which may have hindered our ability to identify other relevant variables. Fourth, we included patients aged ≥15 years because this is the age of adulthood in our country. However, the immune systems of adolescents and adults may differ; further assessments of BKPyVAN in adolescents are needed. We note that there is limited information regarding comparisons of BKPyV DNAuria, BKPyV DNAemia, and immunological factors between adults and children who have undergone KT. Thus, a larger prospective study involving both adults and children is warranted. Fifth, the lack of an IFN‐γ response may be related to either the absence of specific T or NK cells or the impairment of IFN‐γ production. This aspect could be addressed by tests involving tetramers. Finally, the lack of commercial assays could restrict the application of our findings to clinical practice, particularly concerning the use of high donor BKPyV‐specific antibody titers as a marker of recent viral exposure and potentially higher BKPyV viral load in allografts.^31^, ^32^

In summary, we found that initial levels and post‐KT increases in NK, NKT, and VP1‐specific NK cells were greater in KT recipients who exhibited BKPyV DNAuria within 1 year after KT. BKPyV‐specific NK‐ and NKT‐cell immune surveillance could be used to identify patients at risk for post‐transplant viral reactivation. Further studies regarding the underlying mechanisms and clinical impact are needed to confirm this association.

## AUTHOR CONTRIBUTIONS

**Tanaya Siripoon**: Conceptualization; writing—original draft; writing—review and editing. **Nopporn Apiwattanakul**: Methodology; writing—original draft; writing—review and editing. **Pannawat Mongkolrattanakul**: Conceptualization; writing—review and editing. **Chutatip Tongsook**: Methodology; writing—review and editing. **Nattawut Unwanatham**: Formal analysis; writing—review and editing. **Suradej Hongeng**: Methodology; writing—review and editing. **Surasak Kantachuvesiri**: Conceptualization; writing—review and editing. **Jackrapong Bruminhent**: Conceptualization; writing—original draft; writing—review and editing.

## CONFLICT OF INTEREST STATEMENT

The authors declare no conflict of interest.

## ETHICS STATEMENT

The study protocol was approved by the Institutional Review Board of the Faculty of Medicine Ramathibodi Hospital, Mahidol University, Bangkok, Thailand.

## Supporting information

Supporting information.

Supporting information.

## Data Availability

The data that support the findings of this study are available from the corresponding author upon reasonable request.
