# Supplementary material for: Clinical and immunological characteristics for BK polyomavirus‐associated nephropathy after kidney transplantation
Source: Immun Inflamm Dis. 2023 Aug 28;11(8):e956. doi: 10.1002/iid3.956 (PMC10461421; doi:10.1002/iid3.956)
Supplement: Supplementary file 1 — Supporting information. [file IID3-11-e956-s002.docx]

Supplementary Material

**Figure S1.** Study timeline for measuring the BKPyV DNA load in urine and conducting intracellular cytokine staining to assess BKPyV-specific immunity


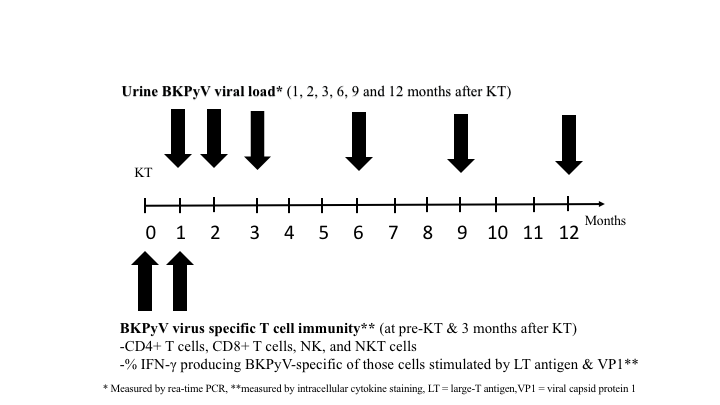


**Figure S2.** Analysis of laboratory-developed intracellular cytokine assays (ICAs) measuring the percentage of IFN-γ-producing CD4^+^ T cells, CD8^+^ T cells, NK, and NKT cells after incubating with LT and VP1

**Figure S2.1** (A-E) represent patient 043 (possible BKPyVAN). **Figure S2.2** (A-E) represent patient 061 (no BKPyVAN). **Figure S2.3** (A-E) represent BK patient 076 (no BKPyVAN). PBMCs were incubated in RPMI 1640 with 5% CO_2_ for 18-24 hours at 37°C. Isolated PBCs were activated by specific peptides (LT or VP1). (A) Demonstrate gating strategy of negative control [side scatter (SSC) vs CD56 (left panel) and CD3 vs CD56 (right panel)] for identification of CD3− and CD3+ CD56+ NK cells. (B) Demonstrate IFN-γ-producing CD3+CD56+ and CD3-CD56+ cells after stimulation by VP1 (B-1) or LT (B-2). (C) Demonstrate gating strategy of side scatter (SSC) vs CD3+ (left panel), CD4+ vs CD8+ (middle panel), and CD4+ and CD8+ producing IFN- γ (right panel)] in negative control. (D) Demonstrate gating strategy of side scatter (SSC) vs CD3+ (left panel), CD4+ vs CD8+ (middle panel), and CD4+ and CD8+ producing IFN- γ (right panel)] after stimulation by VP1. (E) Gating strategy of side scatter (SSC) vs CD3+ (left panel), CD4+ vs CD8+ (middle panel), and CD4+ and CD8+ producing IFN- γ (right panel)] after stimulation by LT.


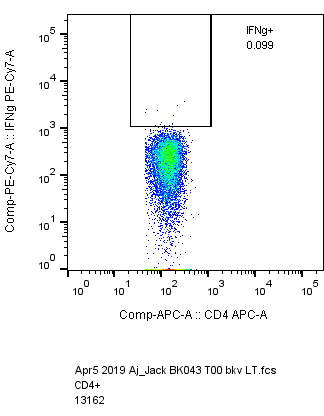

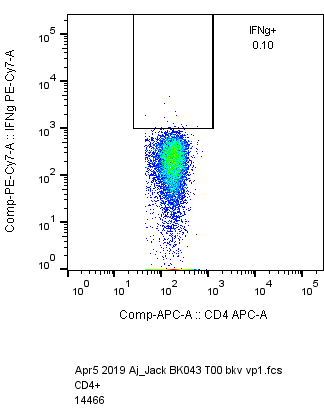


LT

LT

VP1

SSC-A

PE-CD56

PE-Cy7-IFNγ

FITC-CD3

(A)

Negative

control

SSC-A

(B-1)

PE-Cy7-IFNγ

PE-CD56

SSC-A

FITC-CD3

(C)

SSC-A

PE-Cy7-IFNγ

PE-CD56

FITC-CD3

FITC-CD3

APC-eFluor780-CD8

APC-CD4

PE-Cy7-IFNγ

(D)

SSC-A

PE-Cy7-IFNγ

APC-eFluor780-CD8

APC-CD4

FITC-CD3

APC-eFluor780-CD8

(E)

SSC-A

APC-CD4

PE-Cy7-IFNγ

APC-eFluor780-CD8

PE-Cy7-IFNγ

APC-CD4

FITC-CD3

APC-eFluor780-CD8

APC-CD4

PE-Cy7-IFNγ

APC-eFluor780-CD8

PE-Cy7-IFNγ

APC-CD4

Figure S2.1 (A-E) Patient 043 (possible BKPyVAN)


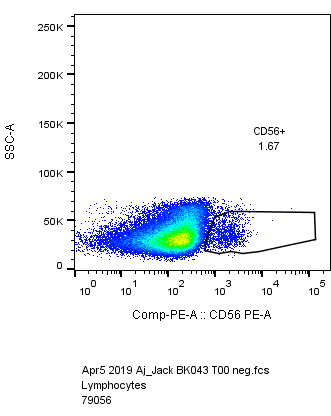

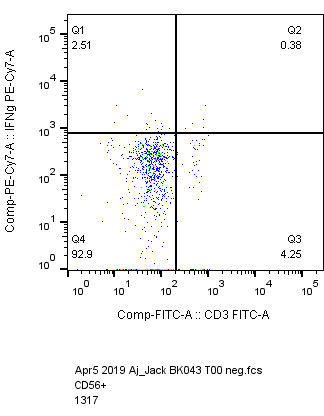

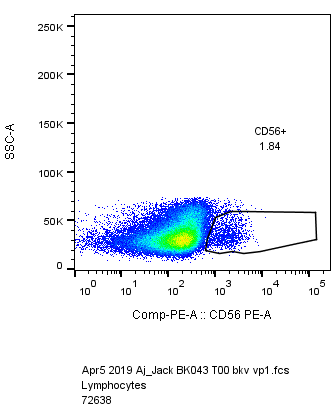

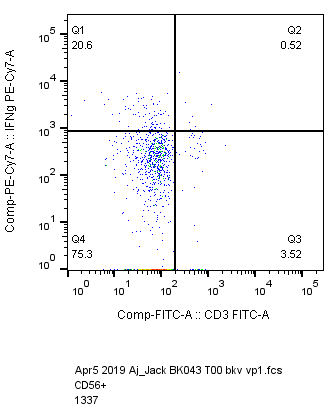

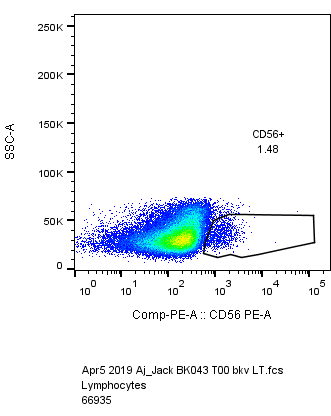

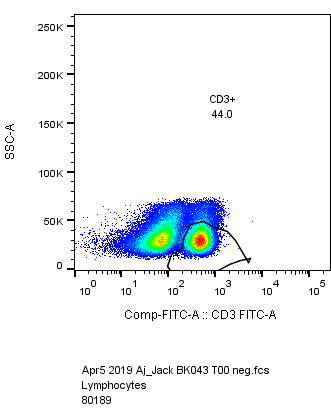

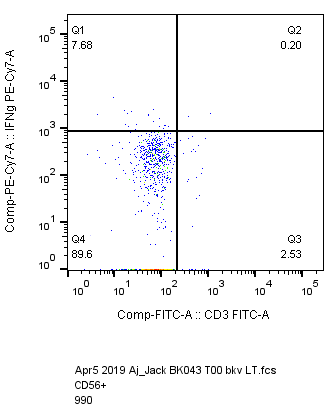

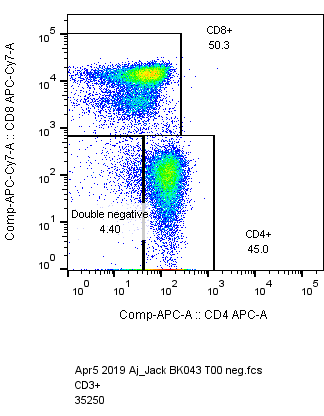

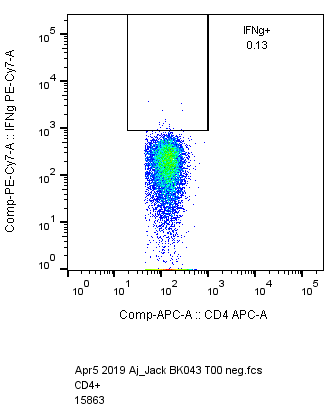

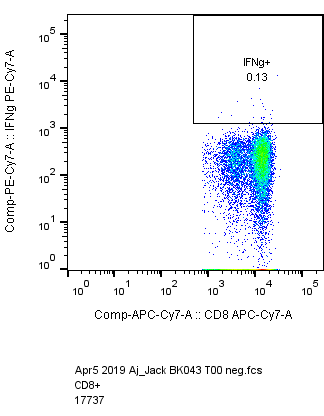

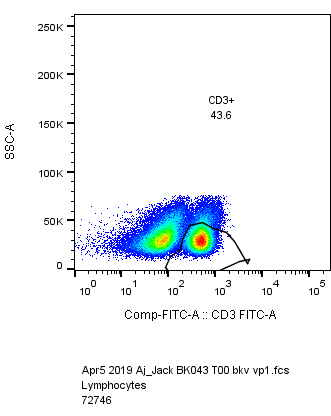

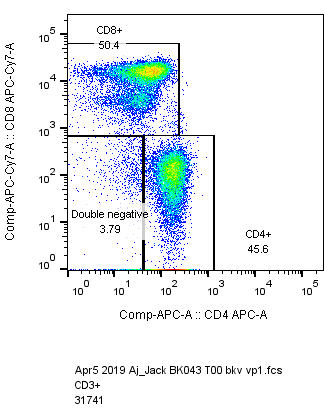

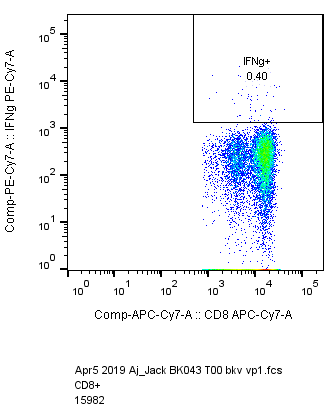

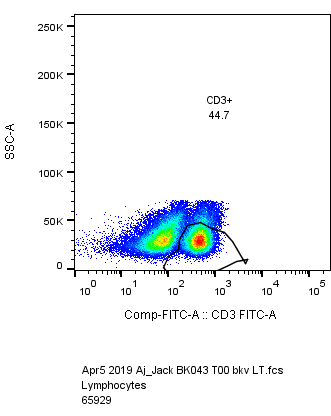

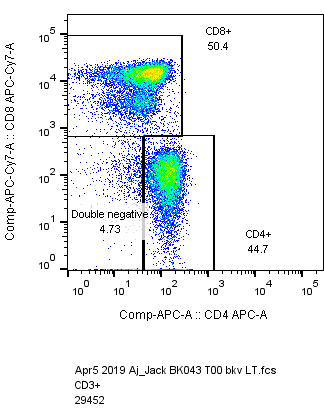

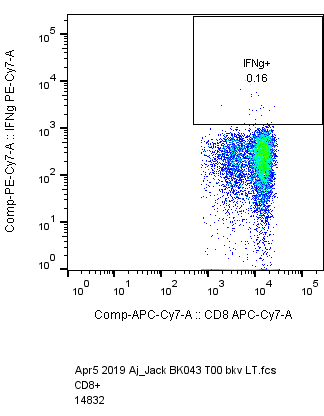


Negative

control

(B-2)

VP1

PE-Cy7-IFNγ


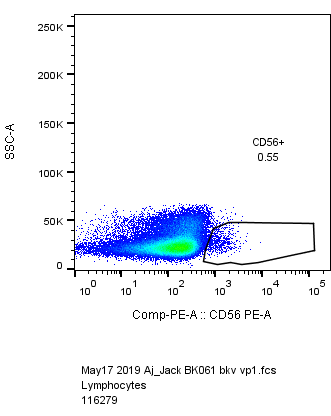

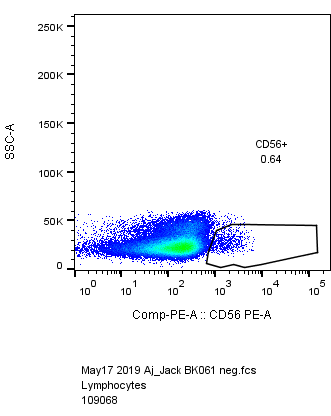

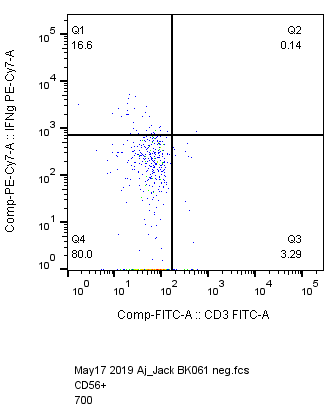

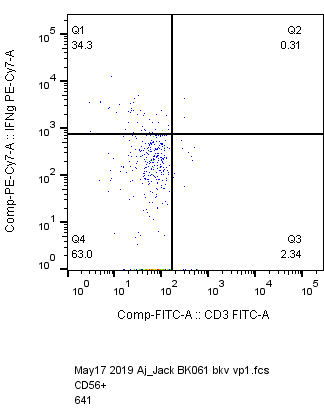

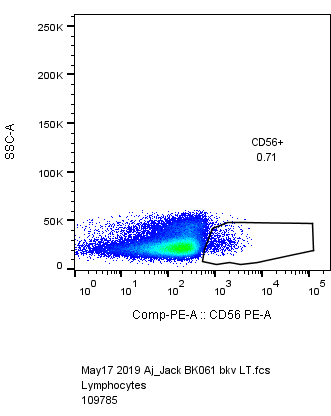

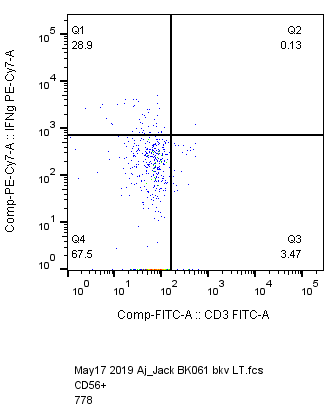

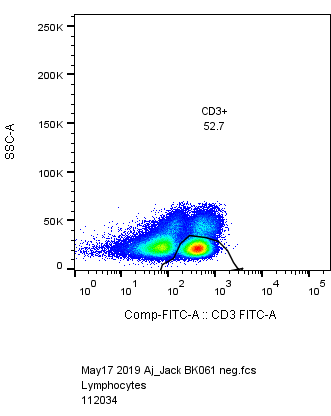

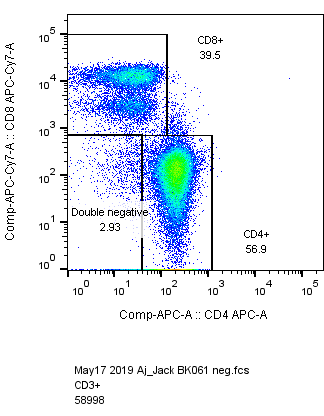

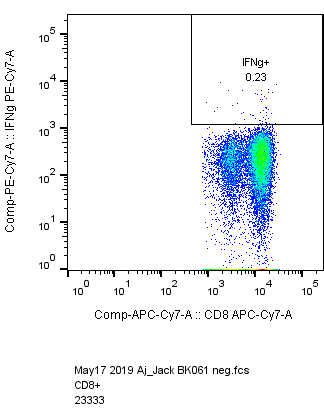

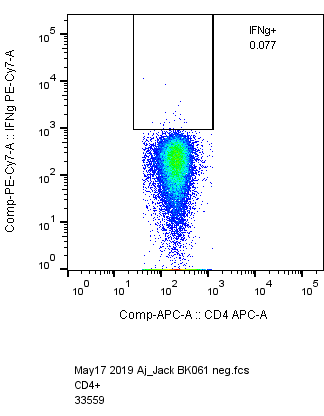

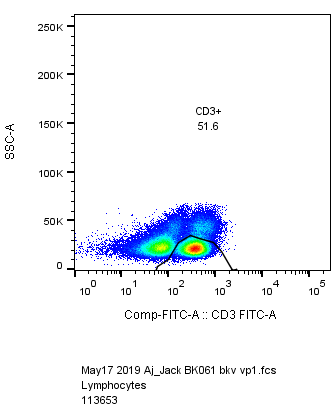

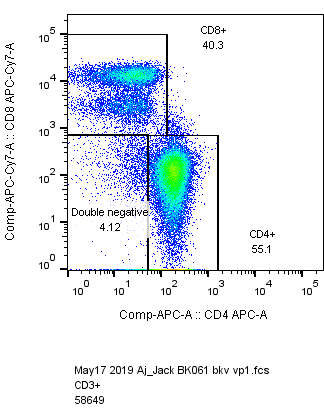

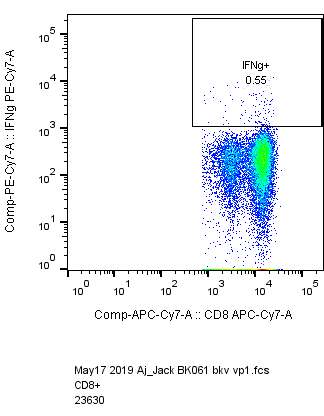

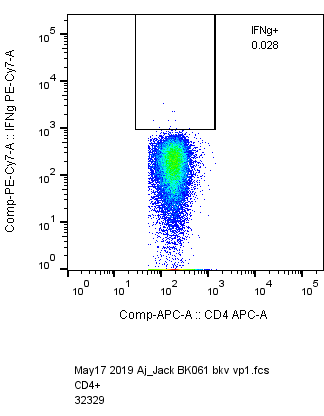

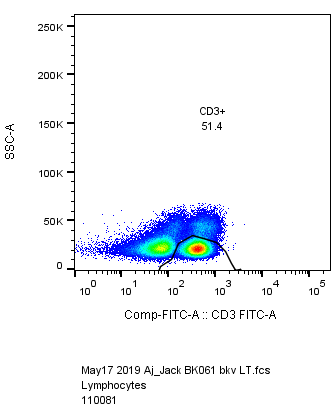

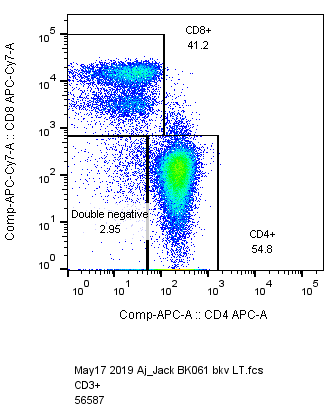

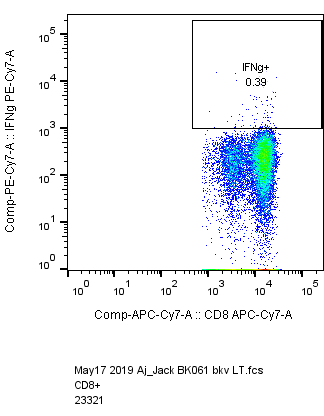

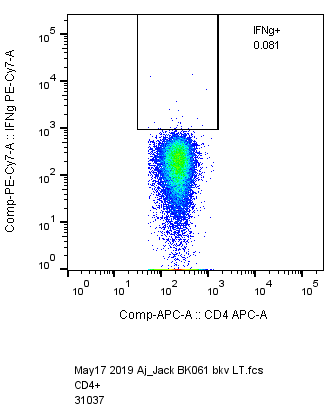


LT

VP1

LTtanaya

SSC-A

PE-CD56

PE-Cy7-IFNγ

FITC-CD3

(A)

Negative

control

SSC-A

(B-1)

PE-Cy7-IFNγ

PE-CD56

SSC-A

FITC-CD3

(C)

SSC-A

PE-Cy7-IFNγ

PE-CD56

FITC-CD3

FITC-CD3

APC-eFluor780-CD8

APC-CD4

PE-Cy7-IFNγ

(D)

SSC-A

PE-Cy7-IFNγ

APC-eFluor780-CD8

APC-CD4

Negative

control

FITC-CD3

APC-eFluor780-CD8

(E)

SSC-A

APC-CD4

APC-eFluor780-CD8

PE-Cy7-IFNγ

APC-CD4

FITC-CD3

APC-eFluor780-CD8

APC-CD4

PE-Cy7-IFNγ

APC-eFluor780-CD8

PE-Cy7-IFNγ

APC-CD4

Figure S2.2 (A-E) Patient 061 (no BKPyVAN)

(B-2)

VP1

PE-Cy7-IFNγ

PE-Cy7-IFNγ


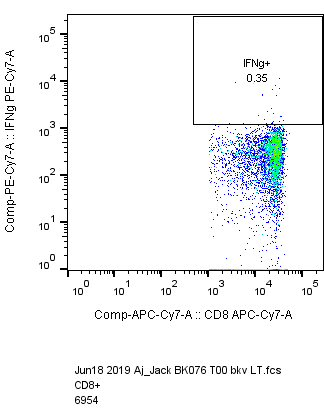

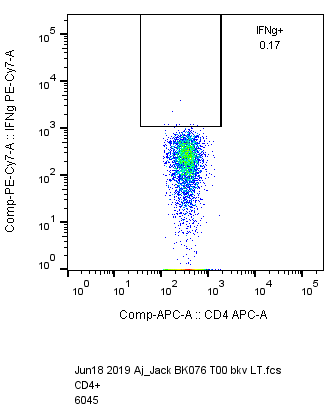

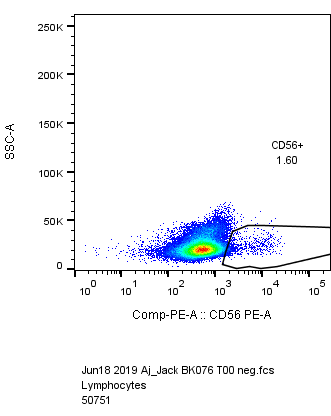

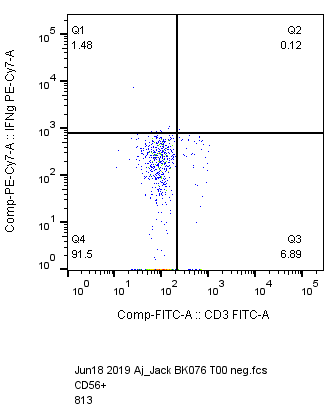

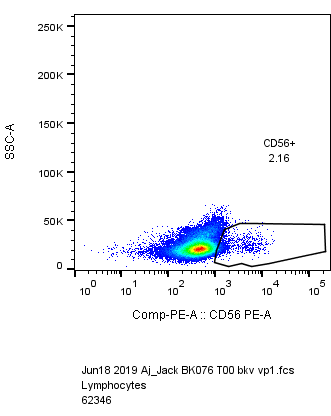

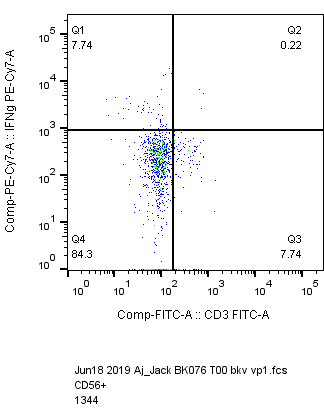

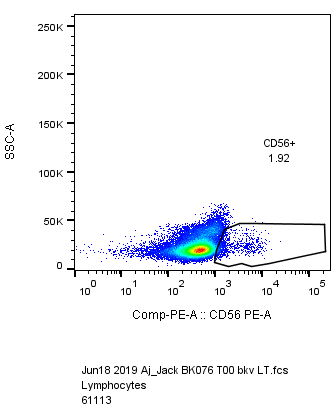

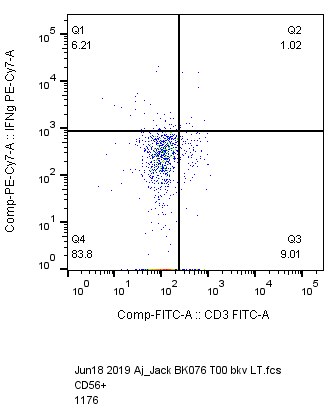

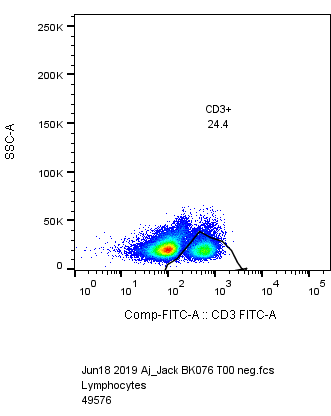

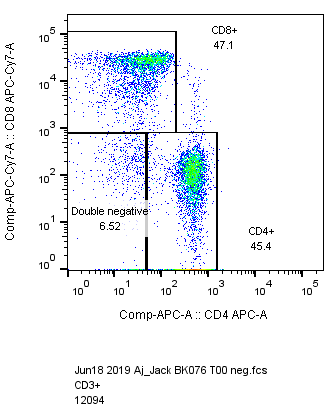

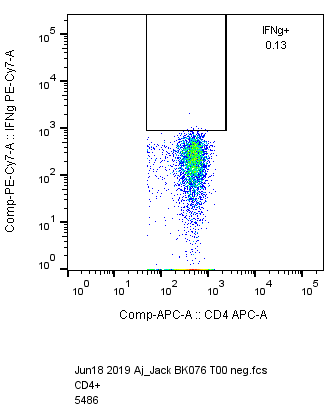

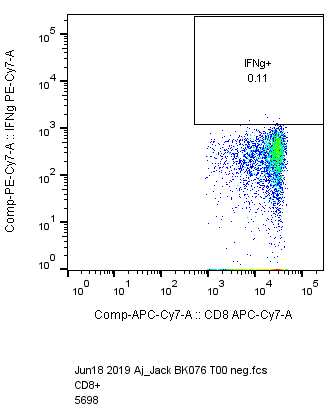

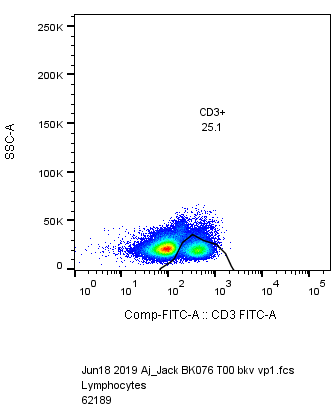

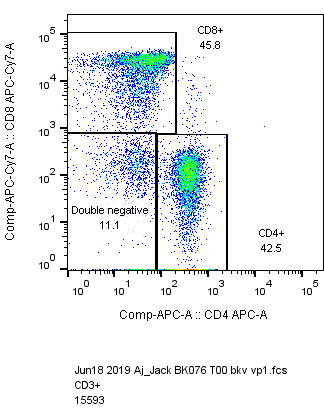

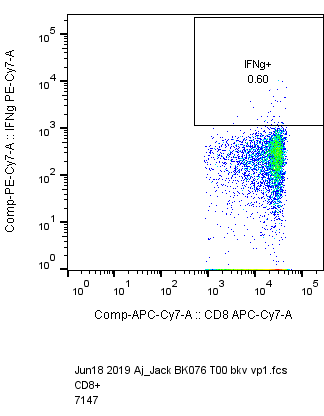

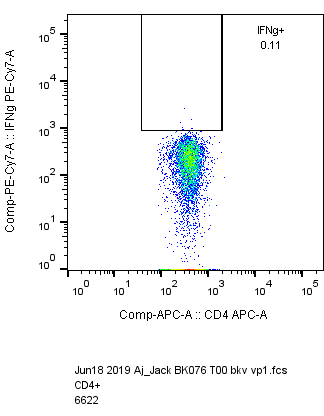


SSC-A

PE-CD56

PE-Cy7-IFNγ

FITC-CD3

(A)

Negative

control

SSC-A

(B-1)

PE-Cy7-IFNγ

PE-CD56

SSC-A

FITC-CD3

(C)

SSC-A

PE-Cy7-IFNγ

PE-CD56

FITC-CD3

FITC-CD3

APC-eFluor780-CD8

APC-CD4

PE-Cy7-IFNγ

(D)

SSC-A

PE-Cy7-IFNγ

APC-eFluor780-CD8

APC-CD4

FITC-CD3

APC-eFluor780-CD8

(E)

SSC-A

APC-CD4

PE-Cy7-IFNγ

APC-eFluor780-CD8

PE-Cy7-IFNγ

APC-CD4

FITC-CD3

APC-eFluor780-CD8

APC-CD4

APC-eFluor780-CD8

PE-Cy7-IFNγ

APC-CD4

Figure S2.3 (A-E) Patient 076 (no BKPyVAN)

PE-Cy7-IFNγ

(B-2)

VP1

LT

Negative

control

VP1

PE-Cy7-IFNγ


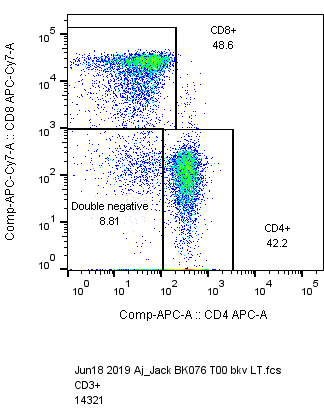

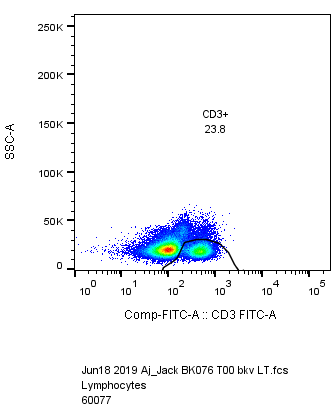


LT

**FigureS3.** Analysis of PE-CD56 staining and control


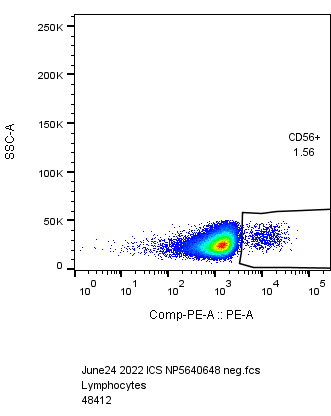

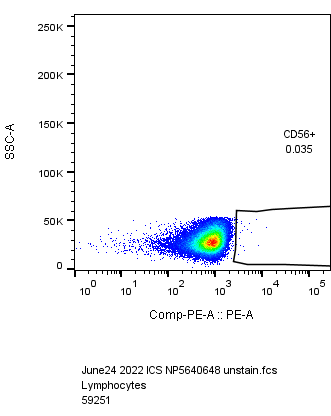


**SSC-A**

**PE-CD56**

**Control**

**+ PE-CD56**

**Table S1.** Definitions of BK polyomavirus (BKPyV) infections based on the American Society of Transplantation Infectious Diseases Community of Practice guidelines published in 2019.

| **Terms** | **Definition** |
| --- | --- |
| Low-level BKPyV DNAuria | Detectable urine BKPyV DNA load but < 7 log10 copies/mL |
| High-level BKPyV DNAuria  (possible BKPyVAN) | Detectable urine BKPyV DNA load > 7 log10 copies/mL |
| Presumptive BKPyVAN | Detectable plasma BKPyV DNA load > 4 log10 copies/mL |
| Proven BKPyVAN | Biopsy-proven polyomavirus-associated nephropathy (viral cytopathic change, inflammatory infiltrates or tubulitis or more than mild interstitial fibrosis/tubular atrophy) |

**Table S2.** Mixed linear effect between immunological factors and time (pre-KT vs. 1-month post-KT) among all KT recipients and those with BKPyVAN

| Factors | All patients (n = 40) | | | BKPyVAN (n = 18) | | |
| --- | --- | --- | --- | --- | --- | --- |
|  | Coefficient | 95%CI | *P*-value | Coefficient | 95%CI | *P*-value |
| %CD4^+^ | 1.527 | −2.272 to 5.325 | 0.431 | −1.253 | −7.650 to 5.145 | 0.701 |
| %LT-specific CD4^+^ | 0.007 | 0.001 to 0.013 | 0.018 | −0.001 | −0.009 to 0.006 | 0.755 |
| %VP1-specific CD4^+^ | less than −0.001 | −0.006 to 0.005 | 0.925 | 0.003 | −0.005 to 0.012 | 0.372 |
| %CD8^+^ | 0.855 | −0.807 to 2.518 | 0.313 | 4.277 | −0.484 to 9.039 | 0.078 |
| %LT-specific CD8^+^ | −0.005 | −0.016 to 0.005 | 0.356 | −0.001 | −0.017 to 0.014 | 0.876 |
| %VP1-specific CD8^+^ | −0.013 | −0.033 to 0.006 | 0.191 | 0.025 | −0.004 to 0.055 | 0.094 |
| %NK | 0.185 | −0.658 to 1.027 | 0.668 | 1.202 | 0.033 to 2.371 | 0.044 |
| %LT-specific NK | −0.269 | −1.030 to 0.493 | 0.489 | −0.307 | −1.503 to 0.911 | 0.621 |
| %VP1-specific NK | −1.007 | −1.934 to −0.080 | 0.033 | 2.602 | 1.083 to 4.121 | 0.001 |
| %NKT | 0.120 | 0.025-0.215 | 0.013 | 0.199 | 0.051-0.348 | 0.008 |
| %LT-specific NKT | 0.294 | −1.861 to 2.449 | 0.789 | −1.671 | −5.331 to 1.990 | 0.371 |
| %VP1-specific NKT | −0.690 | 2.651 to 1.271 | 0.490 | −0.319 | −3.041 to 2.403 | 0.819 |

**Abbreviations:** CI, confidence interval; CD4, CD4 T lymphocyte; LT, large-T antigen; VP1, viral capsid protein-1; CD8, CD8 T lymphocyte; NK, natural killer cell; NKT, natural-killer T cell
